# Synthesis of highly fluorescent Cu/Au bimetallic nanoclusters and their application in a temperature sensor and fluorescent probe for chromium(iii) ions†

**DOI:** 10.1039/c8ra02118j

**Published:** 2018-04-12

**Authors:** Furong Nie, Lu Ga, Jun Ai, Yong Wang

**Affiliations:** College of Chemistry and Enviromental Science, Inner Mongolia Normal University 81 Zhaowudalu Hohhot 010022 China 1257913014@qq.com; Inner Mongolian Key Laboratory for Physics and Chemistry of Functional Materials, Inner Mongolia Normal University 81 Zhaowudalu Hohhot 010022 China imacaj01@163.com; College of Pharmacy, Inner Mongolia Medical University Jinchuankaifaqu Hohhot 010110 China

## Abstract

Bimetallic nanoclusters (BNCs) have attracted great attention due to their cooperative electronic, optical, and catalytic properties. Here, a novel one-step synthetic method is presented to prepare highly fluorescent bimetallic copper–gold nanoclusters (Cu/Au BNCs) in ambient conditions by using glutathione (GSH) as both the reducing agent and the protective layer preventing the aggregation of the as-formed NCs. The resultant Cu/Au BNCs are uniformly dispersed, with an average diameter of 1.5 nm, and it exhibits emission at 450 nm with excitation at 380 nm. Interestingly, the fluorescence signal of the Cu/Au BNCs is reversibly responsive to the environmental temperature, and it shows good sensitivity in the range of 20–70 °C (*F* = −23.96*T* + 3149.2 (*R* = 0.94)). Furthermore, it was found that the fluorescence of Cu/Au BNCs was quenched selectively by Cr^3+^, and a detection method was further developed with detection linear range from 50 nM to 1 mM (*F* = −174.85[Cr^3+^] + 1686.69 (*R* = 0.98)) and high sensitivity (LOD = 10 nM, S/N = 3).

## Abstract 2

The Cu/Au BNCs have been successfully synthesized as a temperature sensor and it successful detection Cr^3+^.

## Graphical abstract

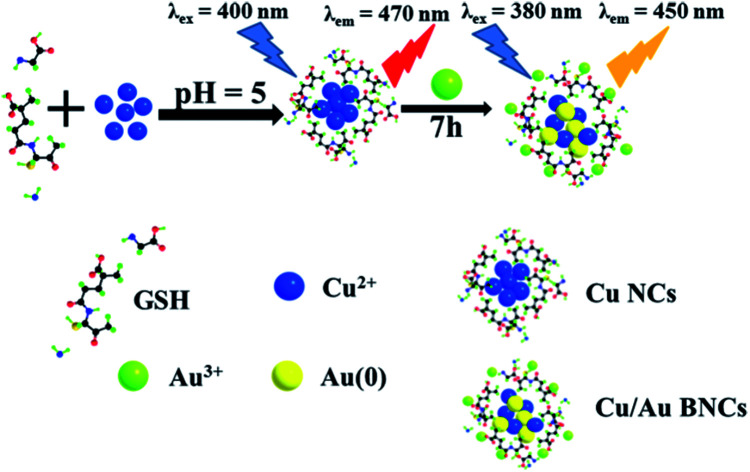

## 1. Introduction

Bimetallic nanoclusters (BNCs) or alloy nanoclusters (ANCs) have attracted more research interest than monometallic nanoclusters (NCs) because of their improved electronic, optical and catalytic properties typically attributed to synergistic effects^1,2^ when two types of atoms having distinct properties are mixed and combined with each other to form electronically and geometrically stable NCs. Great efforts have been made to synthesize BNCs. For example, Yang and co-workers prepared bimetallic Au/Ag NCs as fluorescent probes for Cr^3+^ and Cr^6+^ analysis.^3^ And other researchers did contributions following Table 1.

**Table 1:** Summary of fluorescent bimetallic nanoclusters

Researcher	Bimetallic nanoclusters	Application	Reference
Zhai *et al.*	Au/Ag BNCs	Detection of Hg^2+^	4
Nguyen group	Au/Cu BNCs	Targets to liquid polymer	5
Wang *et al.*	Au/Pd and Pt/Ni NPs	Catalyst for ethanol electro-oxidation	6 and 7
Han *et al.*	Cu/Ag BNCs	Temperature sensor	8
Zhang *et al.*	Ag/Au BNCs	Sensing probes for Hg^2+^	9
Li and co-workers	Pd–M (M = Ni, Ag, Cu)	Catalytic applications	10
Chen group	Ag/Au BNCs	Detection of cysteine	11

Great inspiration was derived from the aforementioned research result that BNCs could also be used to address the issue of weakly fluorescent NCs.

Recently, temperature-sensitive materials have presented remarkable activity because of their application potential to temperature measurement at nanometer scales.^12,13^ In this respect, fluorescence-based temperature sensors have shown great potential, as they operate as “non-contact” tools and offer the dual function of cellular imaging and temperature sensing at the molecular level.^14–16^ Up to date, many promising fluorescent materials, such as quantum dots^17^ and rare earth-doped^18^ and polymeric hydrogel nanoparticles^19^ have already shown a great potential for nanothermometry in biological systems, and especially live cells. Oemrawsingh *et al.* reported that the single emitter fluorescence of Ag NCs increased 5 times when the temperature decreased from 295 to 1.7 K.^20^ Zhou group reported a novel type of dual fluorescence temperature-sensitive DNA-templated silver nanocluster (Ag NCs) pair which contains two pieces of single-stranded Ag NCs in proximity through hybridization and it possess two fluorescence peaks that achieve sensitive variations corresponding to temperature change from 15 to 45 °C.^12^ Wang *et al.* prepared dual-emitting hollow TiO_2_ microsphere could be used as optical thermometry.^21^

Heavy metal contamination of environment has been a paramount global concern in terms of human health and environmental protection over the past decades.^22–24^ Chromium exists universally in nature in two stable states, trivalent Cr^3+^ and hexavalent Cr^6+^,^25,26^ which can sometimes transform into each other. It was reported that chromium in surface water is primarily Cr^3+^, whereas ground-water is mainly Cr^6+^.^26,27^ As Cr^3+^ might be the ultimate reason for toxicity, it is of great significance to explore rapid, simple and selective strategies for the highly sensitive detect it. To date, many methods have been developed for the quantitative determination of chromium, such as electrochemical sensing methods,^28,29^ colorimetric assays,^30,31^ inductively coupled plasma-mass spectrometry,^32–34^ fluorescence detection methods,^35–38^ organic molecule probes detection methods.^39,40^ Among these methods, the fluorescence method is highly focused on because of its ideal sensing performance such as simplicity, short response time and lower cost.

**Scheme 1:**
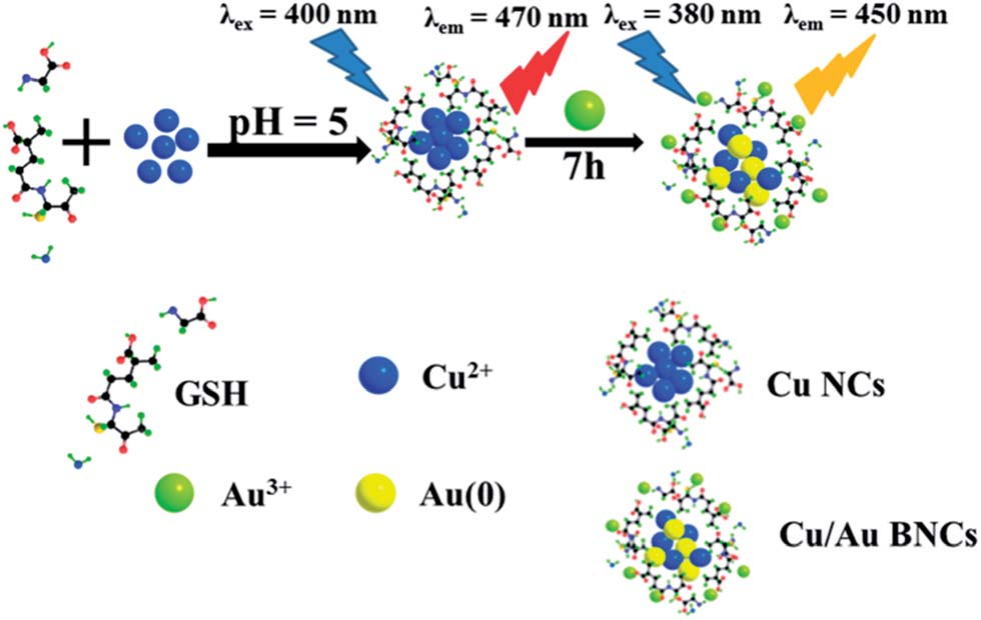
Synthesis of Cu NCs and highly fluorescent Cu/Au BNCs.

In this work, a novel type of high fluorescent Cu/Au BNCs, which presented sensitive fluorescence response to temperature, was prepared. Cu NCs with weak fluorescence was first prepared, followed by the introduction of a certain amount of HAuCl_4_. After few hours, an unexpected strong fluorescence was observed from the as-synthesized Cu/Au BNCs. The synthetic process is illustrated in Scheme 1. To be specific, the fluorescence signal of the Cu/Au BNCs was irreversibly responsive to the temperature in the range of 20–70 °C. And we investigated the response of Cu/Au BNCs to Cr^3+^, and further constructed an analytical strategy. To the best of our knowledge, this is the first study on high fluorescent Cu/Au BNCs to selectively detect Cr^3+^ in aqueous solution. The detection process is illustrated in Scheme S1 in the ESI.†

## 2. Experimental

### 2.1 Materials

Reduced glutathione (GSH, molecular weight of 307.32 g mol^−1^) was purchased from Biotopped, and gold trichloride (HAuCl_4_, molecular weight of 411.8 g mol^−1^) was purchased from Kaima Reagent Co., Ltd. (Tianjin, China). Copper nitrate (Cu(NO_3_)_2_) and sodium hydroxide (NaOH) were purchased from ShengAo Chemicals Co., Ltd. (Tianjin, China). All the other reagents were analytical reagent grade, used without any pretreatment. Deionized water was used in all the experiments.

### 2.2 Cu/Au BNCs synthesis and optimization

The most important reaction parameters, including the mole ratio of Cu(NO_3_)_2_ to HAuCl_4_ and reaction time were investigated to obtain the GSH-stabilized Cu/Au BNCs.

In a standard procedure, a particular weakly fluorescent Cu NCs prepared according to Han group reported work.^8^ For the preparation of GSH-stabilized Cu/Au BNCs, 5 mL of Cu(NO_3_)_2_ solution (20 mM) was added into 5 mL of GSH solution (80 mM) at room temperature, and a white hydrogel was formed because of the coordination between the Cu ions and the functional groups of GSH (*i.e.*, –NH_2_, –COOH, –SH). Subsequently, NaOH solution (1 M) was added dropwise to adjust pH until a light yellow and transparent solution was obtained, and the corresponding pH to about 5. An HAuCl_4_ (1 mL, 80 mM) aqueous solution was introduced into Cu NCs solution, with stirring for 7 h at room temperature. After the reaction, the products were collected by precipitating with deionized water and centrifugation at 5000 rpm. The above purification process was repeated three times, followed by the collection of the precipitate settled at the bottom. The resultant GSH–Cu/Au BNCs were freeze-dried under room temperature and stored in a refrigerator for long-term storage.

### 2.3 Response to temperature

The prepared Cu/Au BNCs solution were incubated at different temperatures for 45 min before a fluorescence test. The temperatures ranged from 20 to 70 °C. The fluorescence spectra were performed on F-4600 fluorescence spectrometer (Hitachi, Japan), with the slit widths of excitation and emission being 10 nm.

### 2.4 Fluorimetric detection of Cr^3+^

The detection of Cr^3+^ in deionized water was conducted with the following procedures. First, 900 μL solution of Cu/Au BNCs and metal ions (100 μL, 1 mM), including Zn^2+^, Cr^3+^, Co^2+^, Pb^2+^, Ni^2+^, Cu^2+^, Mn^2+^, Cd^2+^or melamine solution (100 μL, 1 mM) were mixed thoroughly and reacted at room temperature for a certain period of time, then the fluorescent intensity of each sample was recorded. Second, various concentrations of Cr^3+^ solution (100 μL) were mixed with the aforementioned Cu/Au BNCs solution (900 μL) and incubated at room temperature for a certain period of time. Then the fluorescent spectra were measured at an excitation wavelength at 380 nm and emission wavelength at 450 nm.

### 2.5 Sample characterization

Fluorescence experiments were performed with a Hitachi F-4600 fluorescence spectrometer (Hitachi, Japan) with excitation at 380 nm and emission at 450 nm. Fourier transform infrared spectroscopy (FT-IR) spectra were recorded in the wavelength range of 4000–500 cm^−1^ with a Nicolet Avatar 360 FT-IR spectrophotometer (Thermo Fisher Scientific, USA). To study the morphology and estimate the mean diameter of the resultant GSH–Cu/Au BNCs, transmission electron microscopy (TEM) analyses were conducted on a JEOL-2100F transmission electron microscope (Japan) operating at an accelerating voltage of 200 kV. UltimaIV Powder X-ray diffraction (XRD) patterns were recorded using an X-ray diffractometer (Rigaku, Japan) operating at an accelerating voltage of 40 kV.

## 3. Results and discussion

### 3.1 Optimization of synthetic condition of Cu/Au BNCs and characterization of Cu/Au BNCs

Owing to the presence of a thiol group in its molecular structure, GSH possesses intrinsic metal-chelating properties that ensure the formation of high-affinity metal–ligand clusters.^41,42^ Besides, thiol groups are able to etch larger nanoparticles (NPs) or NCs, reducing their sizes and improving their size monodispersity.^43^ Moreover, the carboxyl and amino groups in the GSH molecule tend to provide a protective layer, ensuring the stability of the fluorescence properties of the Cu/Au BNCs. It has been recently demonstrated that GSH acts as a reducing agent in the synthesis of nanoparticles because of the presence of the amino groups.^43,44^ Therefore, in this study, GSH was chosen to serve as both the protective layer and the reducing agent. By simply mixing GSH with a copper source in aqueous solution, a one-step synthesis of Cu NCs has been realized.^8^ Then, 1 mL of HAuCl_4_ solution with different concentrations of Au^3+^ was introduced into the Cu NCs solution, and the mixture solution was incubated at room temperature, resulting in production of Cu/Au BNCs. The fluorescence intensity of Cu NCs was significantly improved after introduction of HAuCl_4_ (80 mM). The corresponding fluorescence spectra were recorded, as shown in Fig. S1.† Cu^2+^/GSH/Au^3+^ molar ratio, (Cu^2+^/GSH/Au^3+^ = 1 : 4 : 3, 1 : 4 : 4, 1 : 4 : 5 mol mol^−1^) were investigated and the corresponding fluorescence spectra were shown in Fig. S2,† demonstrating the optimal Cu^2+^/GSH/Au^3+^ molar ratio is 1 : 4 : 4. Fig. S3† showed that the Cu/Au BNCs achieved the maximum fluorescence intensity under a vigorous stirring condition for 7 h. A prolonged reaction of 8 h caused the rapid decrease of the fluorescence, indicating the formation of large non-fluorescent nanoparticles.^45^ Furthermore, a vigorous stirring condition was conducive to the growth of Cu/Au BNCs. Therefore, we set the reaction time to 7 h.

**Fig. 1:**
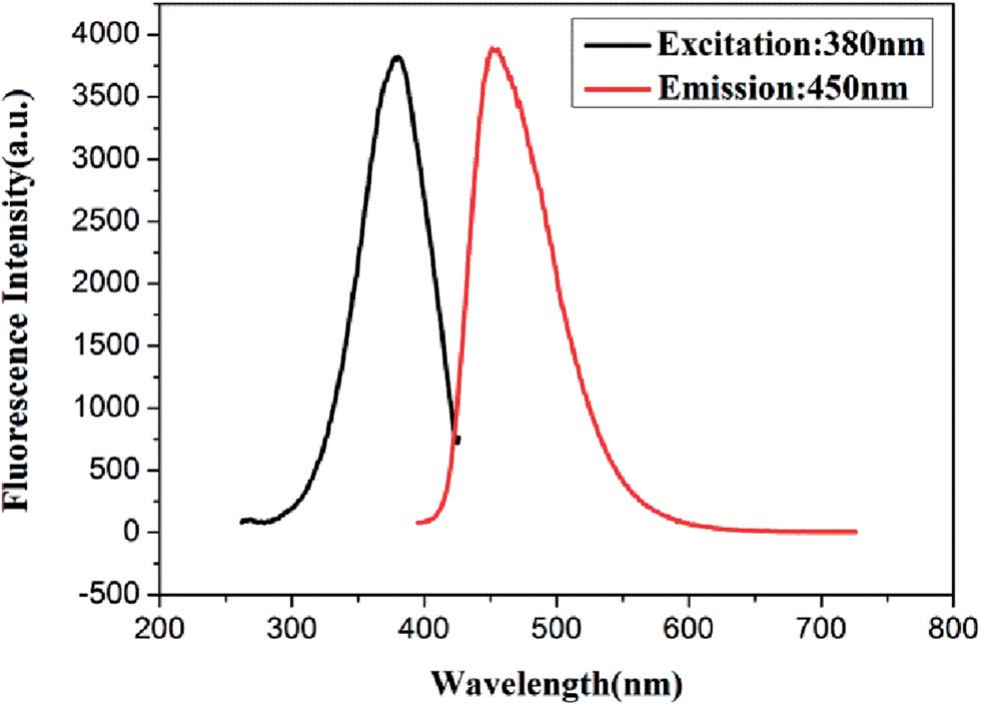
Fluorescence spectra of Cu/Au BNCs (black line) with excitation at 380 nm and (red line) emission at 450 nm.

According to the above experiments, we synthesized the GSH-protected Cu/Au BNCs at room temperature for 7 h with a molar ratio of Cu(NO_3_)_2_ to GSH to HAuCl_4_ equal to 1 : 4 : 4. The fluorescence spectra of the resultant Cu/Au BNCs are shown in Fig. 1. The as-prepared Cu/Au BNCs exhibit good dispersion in aqueous solution with no obvious precipitation, as the protective layer of GSH can prevent the agglomeration of the NCs. In order to further study the optical stability of Cu/Au BNCs, fluorescence spectra analysis was performed, and the results are shown in Fig. S4.† The fluorescence intensity of synthetic Cu/Au BNCs was monitored with time passing. It can be concluded that within 50 days, the change of fluorescence intensity of Cu/Au BNCs are very small. Therefore, Cu/Au BNCs have good stability. The Cu/Au BNCs exhibit emission with a peak at 450 nm and the excitation peak is at 380 nm. The fluorescence excitation band blue shifted from 400 nm to 380 nm, the fluorescence emission band blue shifted from 470 nm to 450 nm, and the fluorescence intensity of Cu/Au BNCs was enhanced instantly about 20-fold, as shown in Fig. S1.†

**Fig. 2:**
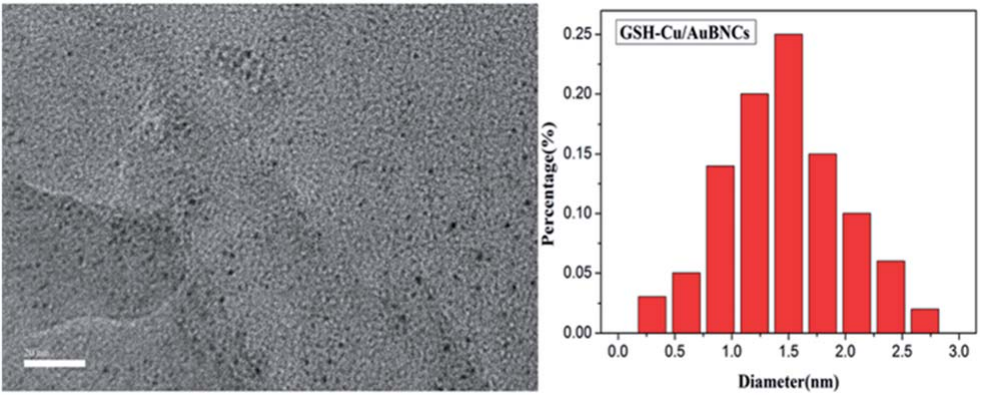
TEM image and size distribution of Cu/Au BNCs.

To directly observe the Cu/Au BNCs, TEM analysis was performed, and the results are shown in Fig. 2. The Cu/Au BNCs are uniformly dispersed and possess an average diameter of approximately 1.5 ± 0.6 nm in the size distribution range of 0.3–2.7 nm, without visible large metal nanoparticles or aggregation. In order to further prove the existence of Cu and Au in the synthesized Cu/Au BNCs, we have proved that Cu and Au are synthesized in the Cu/Au BNCs by the energy spectrum (EDS) test as shown in Fig. S5.†

**Fig. 3:**
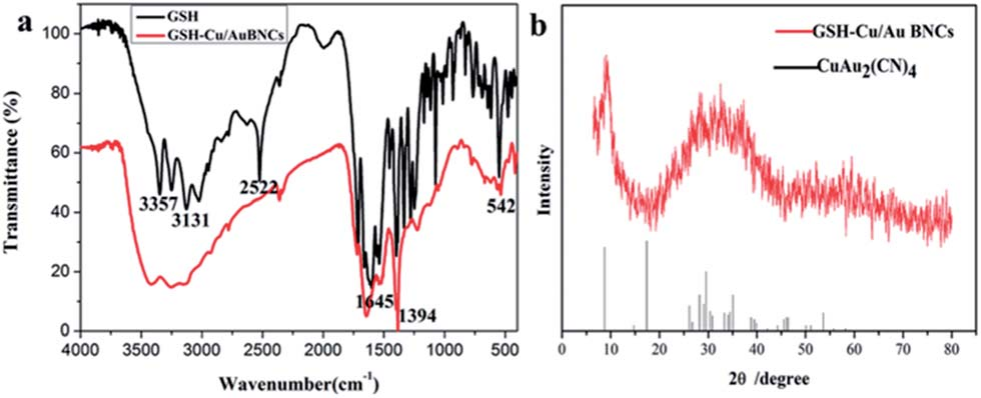
(a) FT-IR spectra of pure GSH, and Cu/Au BNCs. (b) XRD patterns of Cu/Au BNCs (red line) and JCPDS of CuAu_2_(CN)_4_ (black line).

FT-IR spectrograms were recorded to penetrate into the surface properties of the as-prepared GSH and Cu/Au BNCs. As shown in Fig. 3a, in the infrared spectrum of GSH in 3357 cm^−1^ for the N–H stretching vibration, 3131 cm^−1^ for the COOH's C–H stretching vibration, 2522 cm^−1^ for the S–H stretching vibration, 1645 cm^−1^ for the C=O stretching vibration, 1394 cm^−1^ for the C–H stretching vibration and 542 cm^−1^ for the C–C stretching vibration. By comparison, GSH and Cu(NO_3_)_2_ or HAuCl_4_ after reaction, N–H and C–H expansion vibration attenuation, the peak at 2522 cm^−1^ disappeared, indicating the S–H disrupting and the formation of Cu–S and Au–S bonds. The above changes show that GSH has an interaction with Cu(NO_3_)_2_ or HAuCl_4_, and GSH molecules are modified to the surface of Cu/Au BNCs. The powder XRD patterns were used to confirm the formation of Cu/Au BNCs. The Cu/Au BNCs have two large package peaks at 2*θ* = 9°and 2*θ* = 31°, respectively, illustrating. The Cu/Au BNCs are amorphous materials (Fig. 3b).

### 3.2 Fluorescence variation in response to temperature

**Fig. 4:**
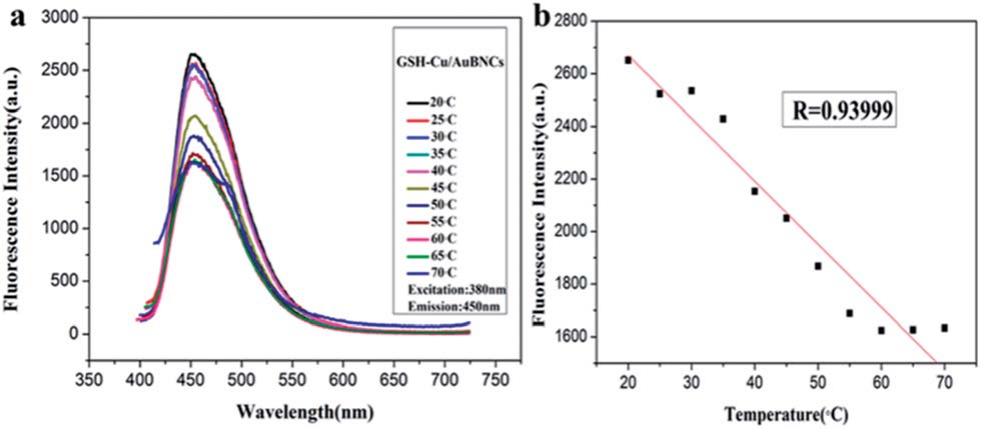
(a) Fluorescence spectra of Cu/Au BNCs with temperature ranging from 20 to 70 °C. (b) The linear relationship between changes of temperature and fluorescence intensity.

To develop the as-prepared Cu/Au BNCs as a temperature sensor, variations of fluorescence spectra in a broad temperature range of 20–70 °C were collected (Fig. 4). When the temperature rises from 20 to 70 °C with an interval of 5 °C, the emission intensity of the Cu/Au BNCs decreased, whereas the emission spectra of the Cu/Au BNCs did not shift within the investigated temperature window (Fig. 4a). This phenomenon might be attributed to thermal agitation of nonradiative processes, the molecule collision frequency and the nonradiative transition rate increase at high temperature, reducing the intensity of the emission from the excited state.^46–48^ The linear relationship between the fluorescence intensity and temperature was within the range of 20–70 °C. As revealed in Fig. 4b, the linear equation was *F* = −23.96*T* + 3149.2 (*R* = 0.94). Conversely, a low temperature is beneficial to increase the fluorescence intensity of the Cu/Au BNCs, but this is an irreversible process (Fig. S6†).

### 3.3 The response of Cu/Au BNCs to Cr^3+^

**Fig. 5:**
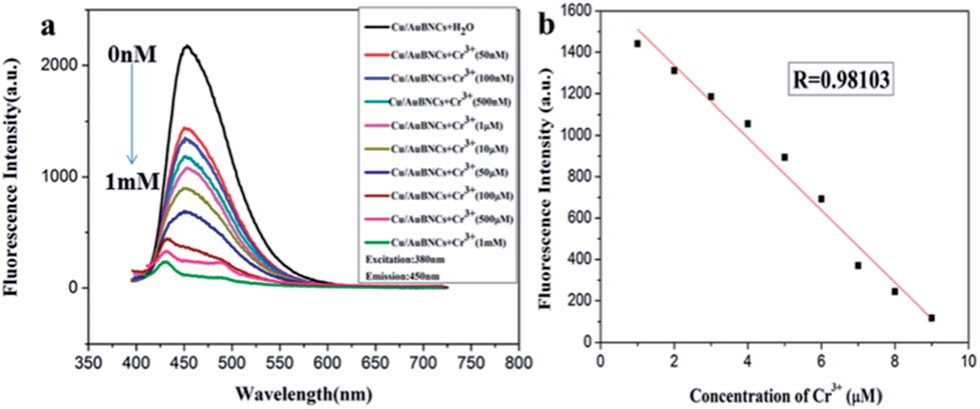
(a) Fluorescence spectra of the Cu/Au BNCs upon the addition of Cr^3+^ increasing from 0 to 1 mM. (b) The linear relationship between the fluorescence intensity and Cr^3+^ concentrations.

Cr^3+^ is an essential trace element for biochemical processes, which plays a vitally important role in insulin action and carbohydrate, lipid and protein metabolism in mammals as enzyme cofactors.^3,26,27^ Lacking Cr^3+^ may increase the risk of illness, such as diabetes mellitus, cardiovascular diseases and fat metabolism disorders.^3^ However, excessive levels of Cr^3+^ are toxic, which is attributed to DNA damage and adverse effects on cellular structures and components, for example, *Shewanella* sp.^3,26^ Herein, the developed fluorimetric method was investigated for the detection of Cr^3+^. Fig. 5a shows the fluorescent spectra of the Cu/Au BNCs at different Cr^3+^ concentrations. With an increase in the Cr^3+^ concentration, the fluorescence intensity of the Cu/Au BNCs gradually decreases, and when the Cr^3+^ aqueous solution was introduced into the Cu/Au BNCs solution, Cr^3+^ could induce the Cu/Au BNCs aggregation, as shown in Fig. S7.† Because of the COOH in glutathione, when Cr^3+^ was introduced into the Cu/Au BNCs protected by glutathione, the OH on the COOH in glutathione will combine with Cr^3+^ to form a gray green chromium hydroxide precipitate. The linear relationship between the fluorescence intensity and Cr^3+^ concentrations within the range of 0.05–1000 μM (Fig. 5b). The linear equation was *F* = −174.85[Cr^3+^] + 1686.69 (*R* = 0.98), and the limit of detection (LOD) of Cr^3+^ was calculated as 10 nM (S/N = 3), which was much lower than Au/Ag NCs-based colorimetric methods.^3^ The results indicate that the as-prepared Cu/Au BNCs can be employed to successfully detect Cr^3+^ in an aqueous medium. Several methods for detected Cr^3+^ are compared in Table 2.

**Table 2:** Summary of several methods for detected Cr^3+^

Researcher	Detection method	Detection range	LOD	Reference
Yang *et al.*	Fluorimetric detection	0.08–6 μM	0.05 μM	3
Zheng *et al.*	Visual chronometric assay	0.03–600 μM	2.7 nM	24
Li *et al.*	Fluorescence recovery assay	0–50 μM	80 nM	26
Nie *et al.*	Fluorimetric detection	0.05–1000 μM	10 nM	This article

**Fig. 6:**
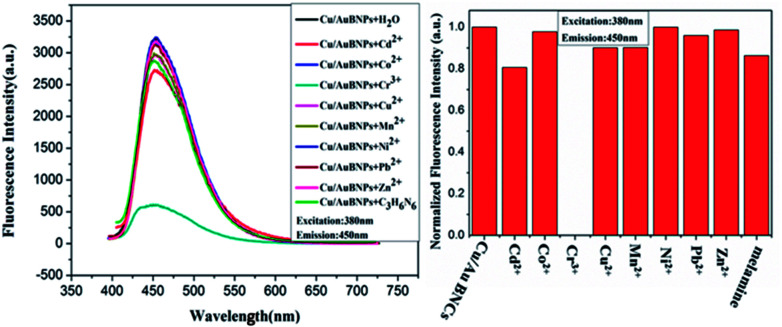
Fluorescence spectra of the Cu/Au BNCs in presence of various common metal ions.

The fluorescence changes of the Cu/Au BNCs after the addition of melamine and different metal ions (Zn^2+^, Cr^3+^, Co^2+^, Pb^2+^, Ni^2+^, Cu^2+^, Mn^2+^, Cd^2+^, respectively) were investigated to test the selectivity of this strategy. Fig. 6 shows that only Cr^3+^ can trigger the fluorescence quenching of the Cu/Au BNCs. In order to further improve the selectivity, all metal ions mixtures were added into the Cu/Au BNCs solution. As expected, it has great selectivity in the detection of Cr^3+^ (Fig. S8†). The result indicated that as-synthesized Cu/Au BNCs sensing prober showed excellent linearity, good stability and high selectivity, exhibited great potential in heavy metal sensing application.

## 4. Conclusions

In conclusion, we present a convenient one-step method to prepare highly fluorescent and stable Cu/Au BNCs using GSH as both the reducing agent and the protective layer. The resultant Cu/Au BNCs show remarkable features including good water solubility and monodispersity, and high fluorescence. It shows good sensitivity and perfect linear response to the variation of temperature, which can be applied for thermal imaging in biological environment. Among common metal ions and melamine, only Cr^3+^ triggered a significant fluorescence quenching of the Cu/Au BNCs. This kind of Cu/Au BNCs-based sensor showed excellent linearity, good stability and high selectivity, and has great potential in heavy metal sensing application.

## Conflicts of interest

There are no conflicts to declare.

## Supplementary Material

RA-008-C8RA02118J-s001
