# Supplementary material for: Synthesis of highly fluorescent Cu/Au bimetallic nanoclusters and their application in a temperature sensor and fluorescent probe for chromium(iii) ions
Source: RSC Adv. 2018 Apr 12;8(25):13708–13. doi: 10.1039/c8ra02118j (PMC9079805; doi:10.1039/c8ra02118j)
Supplement: RA-008-C8RA02118J-s001 [file RA-008-C8RA02118J-s001.pdf]

Electronic Supplementary Material (ESI) for RSC Advances.
This journal is © The Royal Society of Chemistry 2018

Electronic Supplementary Material (ESI) for Analytical Methods. This journal is © The Royal Society of Chemistry 2018

Supporting Information

Synthesis of highly fluorescent Cu/Au bimetallic nanoclusters and their application in a temperature sensor and fluorescent probes for Chromium(III) Ions

Furong Nie, Lu Ga, Jun Ai*, Yong Wang

Inner Mongolian Key Laboratory for Physics and Chemistry of Functional Materials, Inner Mongolia Normal University, 81 zhaowudalu, Hohhot 010022, China.Address here. E-mail: imacaj01@163.com

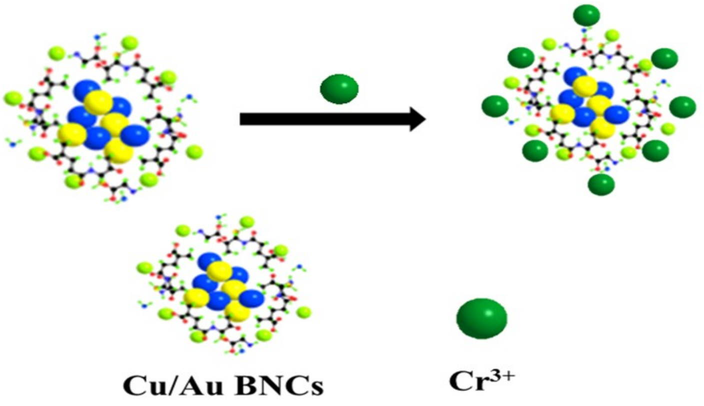

Scheme S1 A schematic representation of Cr^{3+} detection.

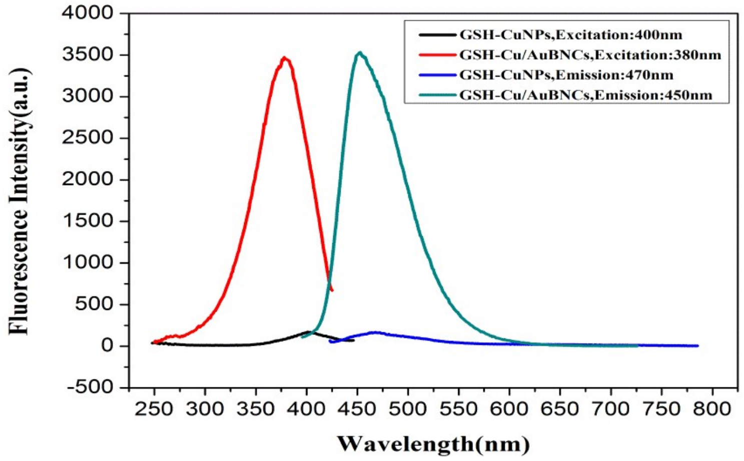

Fig. S1 The fluorescence spectra of the Cu NCs (black line and blue line) with excitation at 400 nm and emission at 470 nm, and Cu/Au BNCs (red line and green line) with excitation at 380 nm and emission at 450 nm.

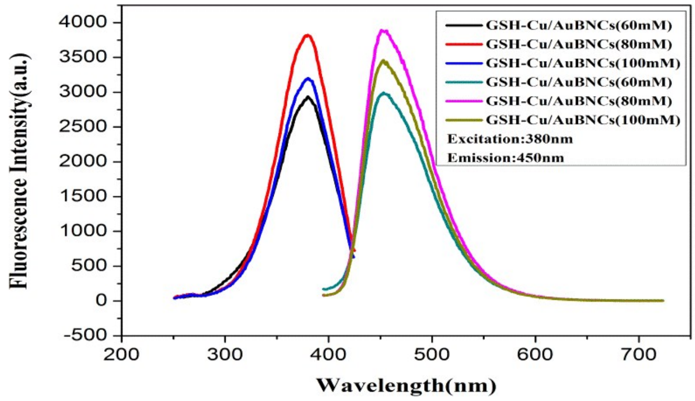

Fig. S2 The fluorescence spectra of GSH- Cu/Au BNCs solution at different Cu^{2+}/GSH/Au^{3+} molar ratios 1:4:3, 1:4:4 and 1:4:5.

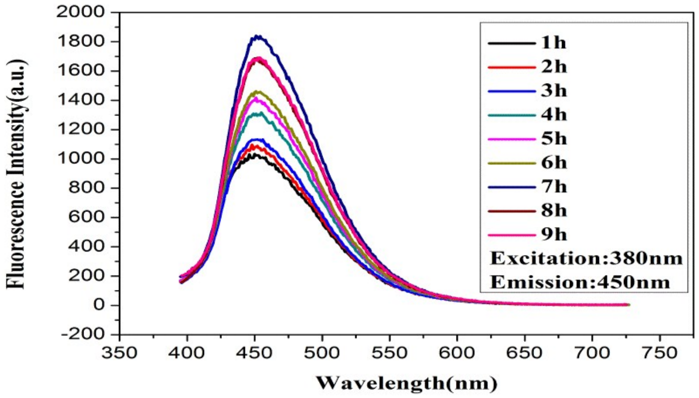

Fig. S3 The fluorescence spectra of Cu/Au BNCs at different stirring times.

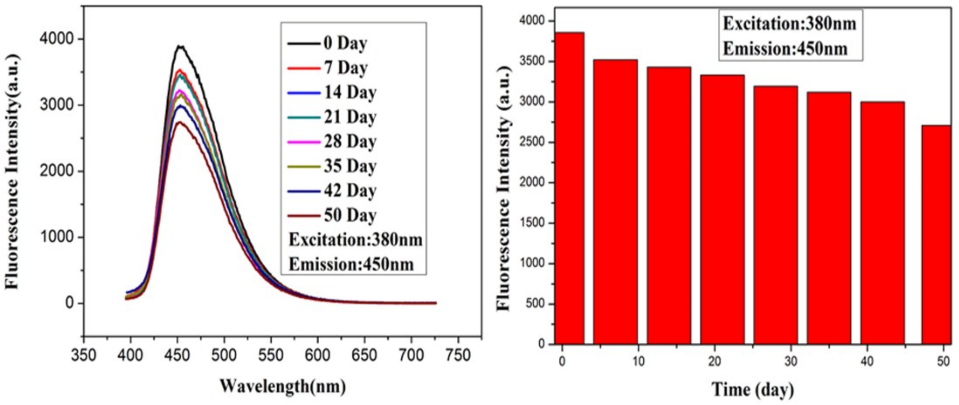

Fig. S4 Fluorescence spectra of the Cu/Au BNCs with time ranging from 0 to 50 day.

The Cu/Au BNCs are deposited on the copper network of carbon support membrane. Therefore, the content of Cu in energy spectrum (EDS) analysis is high.

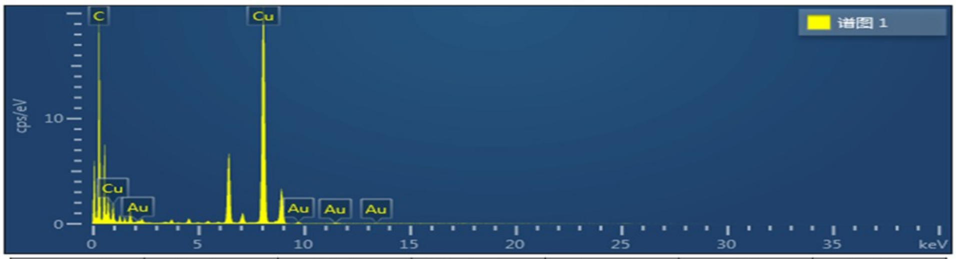

element	line type	k factor	k factor type	absorption correction	wt%	wt% Sigma
C	K line	2.781		1.00	44.08	0.23
Cu	K line	1.230		1.00	54.75	0.23
Au	L line	2.243		1.00	1.17	0.10
total:					100.00	

Fig. S5 EDS image of Cu/Au BNCs (Each sample was deposited on the carbon support membrane copper net for testing).

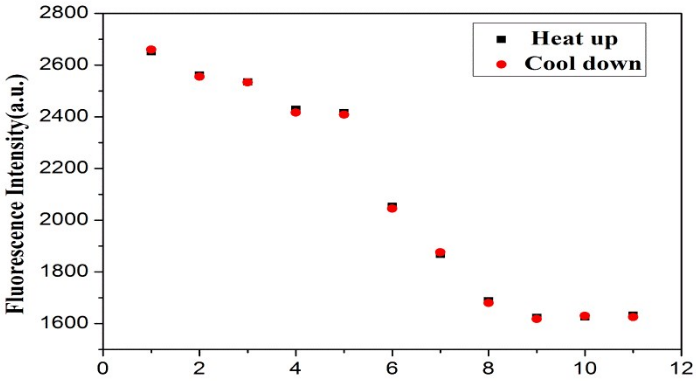

Fig. S6 Fluorescence intensities of Cu/Au BNCs upon 20℃ to 70℃ (black spots) and restore the initial temperature (red spots).

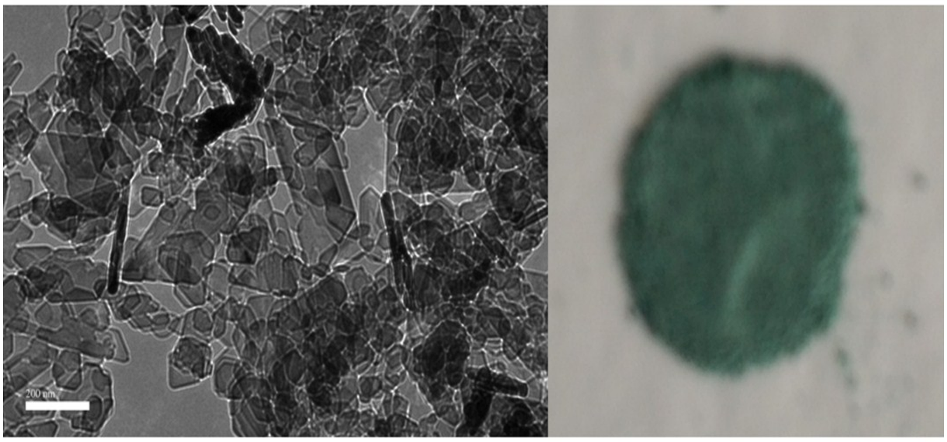

Fig. S7 TEM image of the Cu/Au BNCs detection of Cr^{3+} (left) and grey green sediment Chromium hydroxide (right).

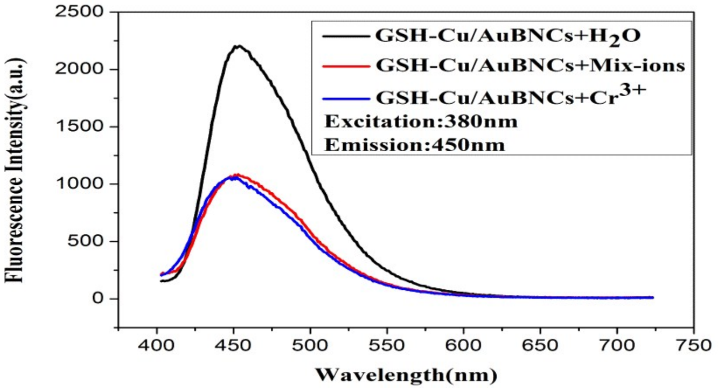

Fig. S8 The fluorescence spectra of the Cu/Au BNCs in presence of various metal ions mixtures (including: Zn^{2+} , Cr^{3+} , Co^{2+} , Pb^{2+} , Ni^{2+} , Cu^{2+} , Mn^{2+} , Cd^{2+}, red line) and the Cu/Au BNCs upon the addition of Cr^{3+} (blue line).
